# Detroit Academy of Medicine

**Published:** 1879-03

**Authors:** E. A. Chapoton

**Affiliations:** Secretary


### DETROIT ACADEMY OF MEDICINE.

November 12th, 1878.

Dr. Noyes reported an interesting case of spontaneous iridocyclitis, followed by sympathetic ophthalmia.

The case, he said, was a confessedly rare one, since sympathetic troubles, as a general rule, are the result of some traumatic injury. Dr. McKenzie, who first described the disease, sympathetic ophthalmia, about thirty-five years ago, says that " lacerated and punctured wounds, involving the iris and ciliary body, are the most active and frequent cause of this disease, and however slight the present symptoms may be, it is one of the most dangerous inflammations to which the organ of vision is exposed; it is also remarkable that the eye which suffers sympathetically is generally more completely destroyed than the one which received the injury." Such is the case with this patient; although the inflammation was not of traumatic origin, the danger in the latter case is just as great. The sympathetic trouble made its appearance about six weeks after the first attack; when the patient came under his observation, the right eye (the first affected) had already passed through the active stage of inflammation; the pupillary margin was firmly adherent to the lens (synechia posterior.) A solution of atropia (6 grs. to ℥j) proved ineffectual in breaking up the adhesions. The sympathetic trouble was fully developed; no adhesions had as yet taken place in the pupil of the left eye; the inflammation, which now involved the whole interior of the globe, was actively combatted by the abstraction of blood from the temple, and by the instillation of atropia, but there appeared little hope of staying the disease; practically, there was little or no vision in the left eye. With the right, which is gradually improving, the patient can now read No. 5 Jaeger's test types.

Unquestionably, in this state of affairs, subsequent treatment is to be directed to improving and preserving the vision of the right, or the eye first affected, and this may call for grave surgical treatment. For instance, should the left eye, besides being useless for vision, offer any hindrance to a complete recovery of the fellow eye, from reflex irritation, it may become necessary to remove it.

Dr. Cleland reported the details of a case of suppurative pericarditis. No one offering to make any remarks upon the subject, Dr. Andrews was called upon.

Dr. Andrews.—I have never met with such cases, and, therefore, can say nothing from my personal observation in the matter. I have had one patient whose pericardium I have always regretted not having tapped, for in the post mortem examination, we found within it more than two gallons of clear serum. But as the dullness extended over the whole front of the chest, the diagnosis of a large consultation of medical men was enlargement of the liver.

Dr. Cleland—Last Tuesday, Dr. Thomas removed a few deciduous teeth from the mouth of a child, but towards morning, Wednesday, the hemorrhage from the cavities became so great that Dr. Hoke was called in to check it. Wednesday afternoon, the blood having started again, with the assistance of Dr. E., Dr. H. etherized the child and cauterized the cavities. Again, that night, hemorrhage set in, even to syncope, and being sent for, I checked the flow by passing a red-hot needle up the cavity. On the separation of the slough, the blood again commenced to flow, and thus it has gone on until this morning, since which time the wound is quiescent. The child nearly died once before from the effects of a nasal hemorrhage. An uncle of the same person died from a hemorrhage from the mucous membranes.

Dr. Walker—A short time ago, I reported the details of an operation, which I had performed two weeks before, of an extirpation of the rectum on account of cancerous disease. As I told you before, I was called to see the patient by Dr. Carsten. The patient stayed in the hospital six weeks after the operation, and departed, feeling very well. He makes use of a quantity of oakum, held in place by a bandage, to replace the function of his lost sphincters. He is now working at his trade in a bakery. I cannot insist too much upon the value of irrigation in such cases, for I believe it is the means of preventing blood-poisoning. Three weeks since, I assisted at another operation of the same kind, and the patient is now doing very well. In this man, however, the sphincters were not removed.

On motion, the meeting was adjourned.

E. A. Chapoton, Secretary.

